# Protection of adipose-derived mesenchymal stromal cells during acute lung injury requires autophagy maintained by mTOR

**DOI:** 10.1038/s41420-022-01267-z

**Published:** 2022-12-05

**Authors:** Yue Hu, Jing Shao, Lanying Shen, Shengchao Wang, Kaiyan Xu, Jiayan Mao, Jian Shen, Wei Chen

**Affiliations:** 1 Key Laboratory of Respiratory Disease of Zhejiang Province, Department of Respiratory and Critical Care Medicine, Second Affiliated Hospital of Zhejiang University School of Medicine, 310009 Hangzhou, Zhejiang China; 2 Cancer Institute of Integrated Traditional Chinese and Western Medicine, Zhejiang Academy of Traditional Chinese Medicine, Tongde Hospital of Zhejiang Province, 310012 Hangzhou, Zhejiang China; 3 Department of Gynecological Oncology, Women’s Hospital, Zhejiang University School of Medicine, 310006 Hangzhou, Zhejiang China

**Keywords:** Cardiovascular diseases, Autophagy

## Abstract

Previous studies suggest that mesenchymal stem cells may represent a promising cellular therapy for acute lung injury (ALI); however, the underlying relevant molecular mechanisms remain unclear. Adipose-derived mesenchymal stem cells (ADSCs) were isolated and characterized by alizarin red staining, oil red staining, and flow cytometry. Lung injury and inflammatory cell infiltration were determined using the Evans blue method, wet/dry weight ratio, and H&E staining. An ELISA was used to detect the concentrations of IFN-γ, IL-2, and TNF-α. Autophagy was detected with an mRFP-GFP-LC3 dual-fluorescence autophagy indicator system, Western blotting, and electron microscopy. We first demonstrated that ADSCs did alleviate the inflammatory responses and tissue damage in lipopolysaccharide (LPS)-induced ALI. Next, we further demonstrated in vivo that autophagy plays a key role in the maintenance of ADSC therapeutic efficacy. In vitro experiments demonstrated that ADSCs co-cultured with alveolar epithelial cells depend on autophagy for significant anti-inflammatory functions. Moreover, the mammalian target of rapamycin (mTOR) is a key regulator of autophagy. Taken together, our findings demonstrate that the effect of ADSC on ALI, especially on alveolar epithelial cells, is dependent on mTOR-mediated autophagy maintenance. The significance of our study for ALI therapy is discussed with respect to a more complete understanding of the therapeutic strategy paradigm.

## Introduction

Acute lung injury (ALI) is a common clinical syndrome involving an acute systemic inflammatory process that causes disruption of the lung endothelial and epithelial layers [1, 2]. Without adequate treatment, ALI could further progress to its most severe form (i.e., acute respiratory distress syndrome [ARDS]). Both ALI and ARDS represent the major causes of morbidity and mortality in severely ill patients. There are approximately 60–70 million annual cases of ALI in China, with a case fatality rate as high as 40–70%; however, the only clinical treatment currently available primarily consists of organ support (e.g., mechanical ventilation) and there is no specific and effective treatment strategy. As a pathological change associated with ALI, diffuse damage to alveolar epithelial cells and endothelial cells of pulmonary capillaries has been observed. Such destruction can lead to disruption of the alveolar barrier and disfigurement of the alveolar structure. ALI symptoms further develop into diffuse pulmonary interstitial emphysema and alveolar edema accompanied by a series of acute inflammatory reactions. Therefore, the key treatment of ALI potentially lies in effectively reducing the extent of inflammatory damage to the alveolar epithelium and endothelium.

Mesenchymal stem cells (MSCs), which provide critical insight for our study, are a type of pluripotent stem cell derived from the mesoderm with the capacity for self-renewal and the potential for differentiation via multiple alternative lineages. MSCs have not displayed an exclusion of, or limitation to, known sources for performing autologous transplantation, or incurred either ethical issues or known problems over immunogenicity and originality in cell transplantation therapy. Thus, we can legitimately infer from their basic properties, that MSCs may be capable of promoting the repair and regeneration of injured cells, regulating immune responses, and improving the anti-inflammatory and anti-infective abilities of tissues. In addition, MSCs may have potential therapeutic effects on bone and cartilage regeneration, myocardial infarction, systemic lupus erythematosus, neurological diseases, liver injury, and ALI. In particular, it has been shown that the loss of follistatin-like protein 1 (FSTL1) can hinder MSC proliferation and differentiation into cartilage [3], SIRT6 can be co-activated with the transcription factor Nrf2 [4] to induce the expression of antioxidant genes and thereby regulate MSC homeostasis. Furthermore, the overexpression of interleukin-10 (IL-10) in MSCs can attenuate the inflammatory response and improve mouse survival in endotoxin-induced ALI [5]. Therefore, gene alterations may provide a justifiable means of determining the regulatory mechanisms and effects of MSCs.

A large number of foundational studies have shown the feasibility of MSC transplantation for the treatment of ALI [6–10]. For instance, MSCs derived from bone marrow can reduce the inflammation of lungs in ALI induced by lipopolysaccharide (LPS), *Escherichia coli*, sepsis, etc., while displaying a general reduction effect on the inflammation response, pulmonary edema, and the bacterial loads in the lung tissue and the lung alveolar lavage fluid [11, 12]. However, such studies have failed to provide decisive evidence concerning whether the protective effect of MSCs is mediated primarily by inhibiting the release of pro-inflammatory factors or by increasing the production of anti-inflammatory factors by T cells, dendritic cells, and natural killer cells. Similarly, there is no evidence concerning whether the soluble cytokines (e.g., prostaglandin E2 [PGE2]) are secreted by neighboring or distant cells to promote enhanced anti-inflammatory effects, enhanced repair of alveolar epithelial and endothelial cells, and reduced alveolar fluid exudation [13]. One fundamental flaw in our belief in an effective therapeutic strategy is the notion that such non-evidenced and insufficiently-clarified molecular mechanisms by which stem cells should regulate the desired anti-inflammatory effects. Therefore, the identification of effectual evidence with increased mechanistic certainty is both essential and critical to elucidate the anti-inflammatory function of MSCs as a therapeutic strategy for ALI.

Autophagy, as a lysosome-dependent degradation pathway that universally exists within eukaryotic cells may be involved in ALI due to its important role in maintaining the recycling of cellular material and homeostasis [14]. When autophagy as a universal suspect occurs, the activation of type III PI3K, which is dependent on the inactivation of mTORC1 by the cellular target of the rapamycin complex, is required for the formation of autophagy precursors. LC3 is an autophagosome marker that plays a key role in the growth and elongation of the autophagic membrane. Moreover, LC3 interacts with extracellular signal-regulated kinase (ERK) [15] to regulate various cellular properties. Studies have shown that the regulation of stem cell functions by autophagy has created an area of uncertainty to respond to the local context challenge over the presence or absence of its role in respiratory diseases, specifically regarding the underlying mechanistic processes. Our group previously summarized the regulatory and molecular mechanisms of autophagy in lung tissue cells and found that autophagy plays different regulatory roles in different cells and models [16]. Echoing a recent finding that the deletion of the autophagy proteins ATG7 or ATG5 in myeloid cells spontaneously induces lung inflammation [17], our group found that the overexpression of the autophagy proteins LC3b or ATG5 in airway epithelial cells significantly alleviated LPS-induced inflammation. Such results extend our universal suspicions, suggesting that there is no easy solution to this issue.

Recent studies have shown that autophagy is closely related to processes such as MSC proliferation, differentiation, anti-inflammation, and self-renewal [18–20]. Inhibition of cell autophagy can promote MSC cell death by inducing intracellular reactive oxygen species (ROS) accumulation and DNA damage, activating the mTOR signaling pathway, and disrupting mitochondrial function [21–23]. However, the effect and mechanism of autophagy on MSC protection against ALI remains unclear, and the molecular mechanism underlying the ADSC-regulated anti-inflammatory effect requires further elucidation.

Our previous study demonstrated that activation of MTOR in the epithelium promotes LPS-induced ALI [24]. Therefore, we further hypothesize that the increase of the cellular autophagy level in ADSC harbors a key regulatory role for the anti-inflammatory effects. In this study, our results demonstrate: (1) that adipose-derived mesenchymal stem cells (ADSCs) alleviate inflammatory responses and tissue damage in lipopolysaccharide (LPS)-induced ALI; (2) autophagy does, like a functional harbor, contribute a key role in the maintenance of the therapeutic efficacy of ADSCs in vivo; and (3) ADSCs co-cultured with alveolar epithelial cells in vitro are dependent on autophagy for their significant anti-inflammatory functions, and mTOR is a key regulator of autophagy. Together, the findings of this study provide novel targets and an experimental basis for the treatment of ALI.

## Results

### ADSC characterization

**Fig. 1:**
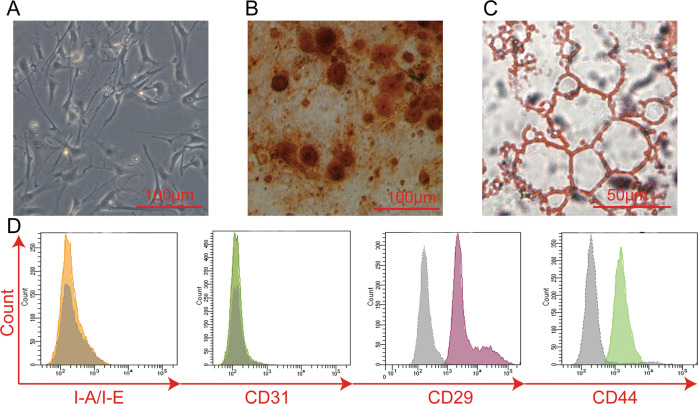
Isolation and characterization of adipose-derived mesenchymal stem cells. Stem cell profiles of in vitro cultured ADSCs from mice were evaluated through differentiation assays. **A** The ADSC bright-field photos were taken using a light microscope. **B**, **C** Stem cell pluripotency was evaluated by culture osteogenesis and adipogenesis under differentiation stimuli and posterior staining with (**B**) Alizarin Red and (**C**) Oil Red. Histograms for stem cell marker expression (**D**) in ADSCs (CD44, CD29 positive; I-A/I-E, CD31 negative) were obtained with a cytometric analysis. Gray plots represent the negative control, while other colored plots represent the surface marker antibody‑stained cells.

ADSCs isolated from subcutaneous mouse inguinal adipose tissue were cultured in vitro for expansion. Cultured cells exhibit typical fibroblast morphology under a light microscope (Fig. 1A). ADSCs were further cultured under differentiation stimuli for osteogenesis and adipogenesis to determine stem cell pluripotency. Differentiated adipocytes and osteocytes were demonstrated through Alizarin Red (Fig. 1B) and Oil Red staining (Fig. 1C). Under appropriate stimuli, the ADSCs exhibited the potential for adipocyte and osteocyte differentiation as demonstrated through Alizarin Red (Fig. 1B) and Oil Red staining (Fig. 1C). Stem cell pluripotency was also detected by cell flow cytometric staining. the cells expressed the typical MSC markers, CD44 and CD29, and stained negative for I-A/I-E and CD31 (Fig. 1D). Together, these results indicate that ADSCs were generated with the correct phenotype and function.

### ADSC ameliorates LPS-induced ALI

**Fig. 2:**
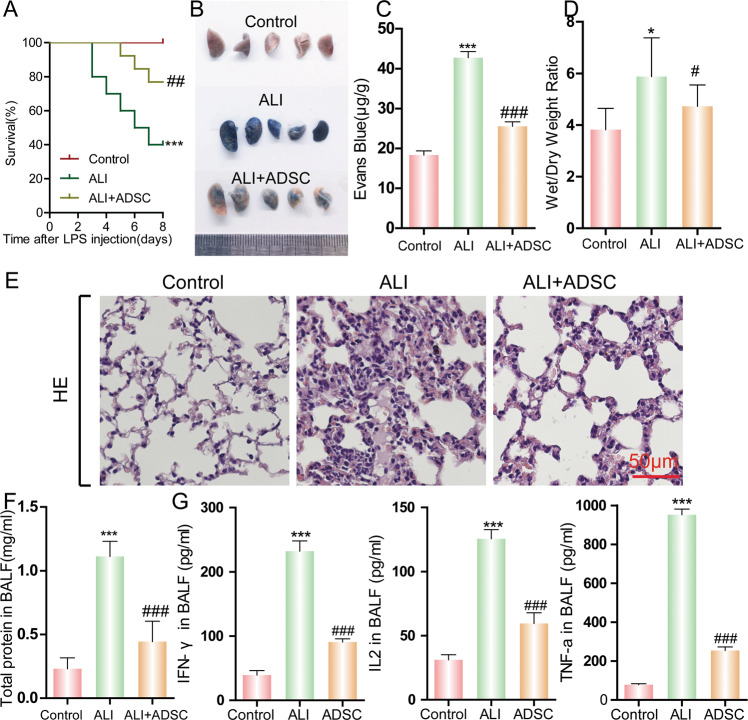
The therapeutic efficacy of ADSCs in LPS-induced ALI in mice. ALI in mice was induced by the airway administration of LPS. ADSCs were injected into the mouse tail vein to study its protective effect on ALI. **A** Mouse survival was monitored for 8 days (20% body weight loss). ***P < 0.001, ^##^*P* < 0.01. (determined by a log-rank (Mantel–Cox) Test). Acute lung tissue injury was evaluated by (**B**) Evans blue staining and (**C**) quantification. Evans blue concentration presents micrograms of Evans blue dye per gram of lung tissue. **D** The wet/dry weight ratio of lung was determined. **E** Pathological changes in the tissue were observed via H&E staining. **F** The total protein concentration was measured. **G** Concentration of inflammatory cytokines IFN-γ, IL-2, and TNF-α, was detected by ELISA. Data are shown as means ± SD (*n* = 10), *P < 0.05, ***P < 0.001 vs control; ^#^*P* < 0.05, ^###^*P* < 0.001 vs ALI. (determined by unpaired *t* test or one-way ANOVA with Tukey comparisons.).

To evaluate the effect of ADSCs for the treatment of ALI, we established a mouse model by administrating LPS via the airway. To study the therapeutic potential of ADSCs, PBS or in vitro cultured cells were injected into the tail vein of ALI mice. LPS-induced ALI led to only 40% survival of the mice in 8 days, whereas the administration of ADSC significantly increased this rate to 80% (Fig. 2A). This finding reveals the therapeutic role of ADSCs in LPS-induced ALI. Evans blue staining revealed severe vascular leakage following LPS challenge in the mouse lungs, and an ADSC injection demonstrated a significant protective effect against lung tissue damage (Fig. 2B, C). LPS challenge also significantly increased the level of capillary leakage in the ALI group of mice. A measurement of the lung wet/dry weight ratio also confirmed that ADSCs suppressed lung capillary leakage in LPS-induced ALI (Fig. 2D). Histological conditions reflected by the H&E staining results also showed consistent results (Fig. 2E). In the mouse control group, the normal lung structure remained intact, the alveolar cavity was clear, and there were no exudates. The alveolar wall did not display any thickening, the alveolar interstitium had no inflammatory cell infiltration, and the structure of the alveolar septa was uniform and consistent. In the LPS-induced ALI group, obvious structural destruction of the lung tissue was observed. The size of the alveoli was different and the alveolar wall displayed significant edema and widening. The alveolar space was collapsed with the formation of emphysema. While LPS administration caused severe histological tissue damage, patches of injury and the presence of inflammatory infiltrates and vascular congestion were all significantly alleviated in the ADSC treatment ALI group compared with the non-treated ALI group. An ADSC injection significantly relieved the extent of lung tissue damage and immune cell infiltration. This suggests that ADSCs may relieve LPS-induced ALI via its anti-inflammatory role and protective effect on tissue injury.

### ADSC has anti-inflammatory effects on LPS-induced ALI

Since the injection of ADSC significantly relieved both lung tissue damage and immune cell infiltration, we hypothesized that ADSCs might play an anti-inflammatory role. Previous data showed that LPS challenge produced a significant increase in capillary leakage in ALI mice. To evaluate the inflammatory condition in the lungs of LPS-induced ALI mice with or without the administration of ADSC, BALF was collected from the mouse lungs, and the concentrations of total protein and inflammatory cytokines were measured. The BALF concentrations in the ALI mouse lung increased to a high level compared with the control, whereas the administration of ADSCs significantly suppressed an increase in the protein concentration induced by LPS (Fig. 2F). The ELISA results also showed that concentrations of IFN-γ, IL-2, and TNF-α were elevated under LPS challenge (Fig. 2G). An injection of ADSCs suppressed the production of these cytokines, suggesting its potential anti-inflammatory effect. We conclude that ADSC has a therapeutic function on LPS-induced ALI via its anti-inflammatory role and protective effect on tissue injury.

### The autophagy pathway is important for the therapeutic efficacy of ADSCs on mouse ALI

Recent studies have shown that autophagy plays an important role in the cellular processes of MSCs, including self-renewal, proliferation, differentiation, and anti-inflammation. Recent studies have shown that MSCs maintain a higher basal level of autophagy, which is significantly reduced during differentiation [18]. High levels of autophagy in MSCs provide a cell survival signal [21–23], and also promote the secretion of vascular endothelial growth factor for improved vascular repair capacity, reduce Th17-associated pro-inflammatory factor production, and enhance autoimmune regulatory effects [19, 20].

**Fig. 3:**
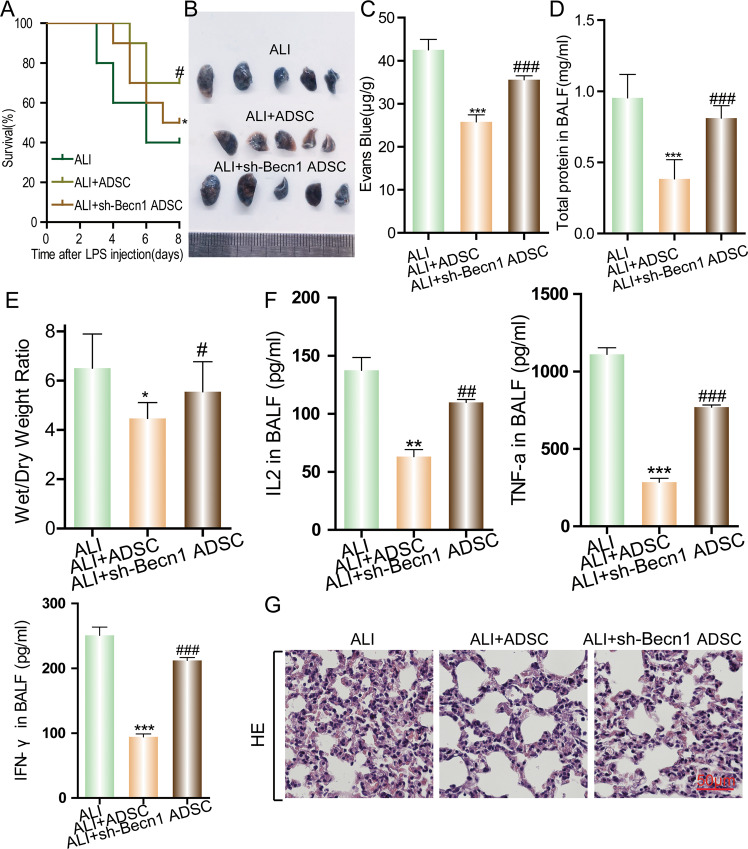
Interference of the autophagic pathway in ADSCs decreases its therapeutic efficacy on mouse ALI. Next, we aimed to study the effects of the autophagic pathway in ADSCs on the therapeutic efficacy of ALI in mice with LPS-induced ALI. To this end, the autophagic pathway was interfered via shRNA targeting Becn-1 in ADSCs as a therapy. ALI in mice was induced by LPS administration via the airway. ADSCs infected with or without the sh-Becn-1 virus were injected into the mouse tail vein to study its protective effect to ALI. **A** Mouse survival was monitored for 8 days (20% body weight loss). *P < 0.05, ^#^*P* < 0.05 (determined by a log-rank (Mantel–Cox) test). Acute lung tissue injury was evaluated by Evans blue staining (**B**) and quantification (**C**). Evans blue concentration presents micrograms of Evans blue dye per gram of lung tissue. **D** Alveolar lavage fluid was collected from the lungs of ALI mice to measure the total protein concentration. **E** The lung wet/dry weight ratio was determined. The concentration of the inflammatory cytokines IFN-γ, IL-2, and TNF-α (**F**) was detected by ELISA, and **G** pathological changes in the tissue were observed using H&E staining. Data are shown as means ± SD (*n* = 10), *P < 0.05, **P < 0.01, ***P < 0.001 vs ALI; ^#^*P* < 0.05, ^##^*P* < 0.01, ^###^*P* < 0.001 vs ALI + ADSC. (determined by unpaired *t* test or one-way ANOVA with Tukey comparisons.).

We hypothesize that autophagy in ADSCs also plays an important role in its therapeutic efficacy against ALI. shRNA targeting Becn-1, one of the key proteins in autophagic pathway, was used to inhibit autophagy in ADSCs. The protective efficacy of normal ADSCs and Becn-1 knockdown ADSCs were compared in an ALI mouse model. While ADSC administration significantly increased the survival rate of ALI mice from 40 to 70%, Becn-1 knockdown ADSCs only increased the rate to 50% (Fig. 3A). These results indicate that autophagy in ADSCs plays an important role in the maintenance of its therapeutic effect on LPS-induced ALI. The pathological conditions in the lungs of the mice were further analyzed using Evans blue staining, The protein concentration in the BALF and lung wet/dry weight ratio were also measured (Fig. 3B–E). The results confirmed that the ADSCs with a Becn-1 knockdown had lost protective efficacy against tissue damage in ALI. An ELISA of IFN-γ, IL-2, and TNF-α in the alveolar lavage fluid of the mice further revealed that the Becn-1 knockdown ADSCs could no longer provide an anti-inflammatory function as they provided minimal suppression of cytokine secretion (Fig. 3F). The histological staining showed that although there were patches of tissue damage, the presence of infiltrates and vascular congestion were significantly reduced in the lung tissue of ADSC-treated ALI mice. In contrast, the Becn-1 knockdown ADSCs have only a slight protective effect (Fig. 3G). All the results indicate that autophagy plays a key role in maintaining the inhibitory effect of ADSCs on lung inflammation and tissue damage.

### Autophagy is required for the anti-inflammatory function of ADSCs

**Fig. 4:**
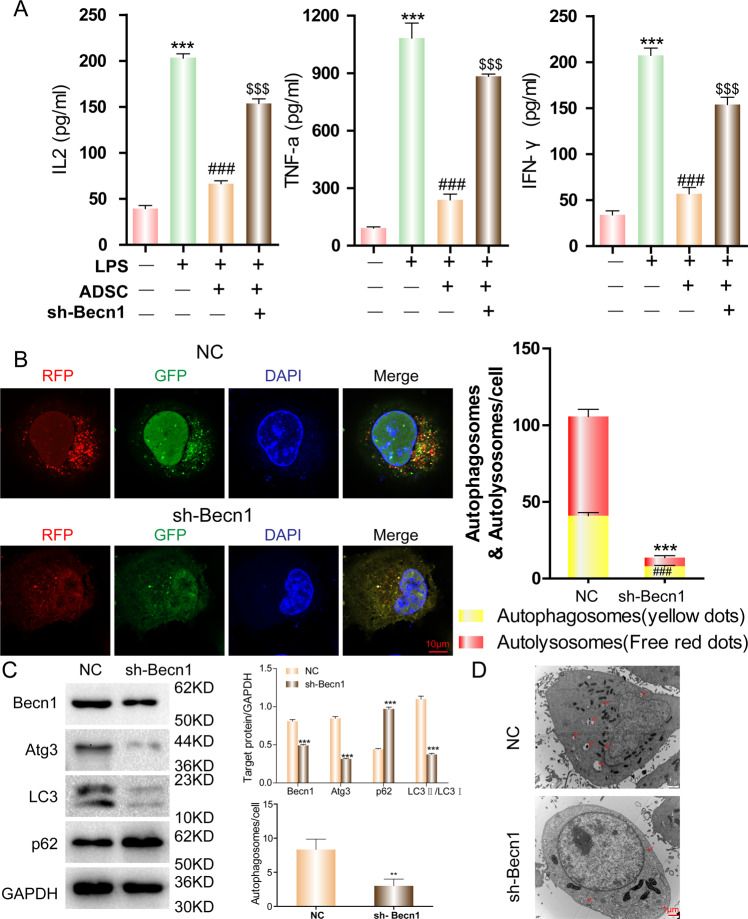
The role of autophagy on the anti-inflammatory function of ADSCs. ADSCs were cultured in vitro, and becn-1 expression in ADSCs was knocked down by shRNA. Alveolar epithelial cells (MLE-12) were cocultured with ADSCs using a Transwell system. The MLE-12 cells were subsequently stimulated with LPS. **A** Inflammatory cytokines IL-2, TNF-α, and IFN-γ in the supernatants of different groups were detected by ELISA. ***P < 0.001 vs control group; ^###^*P* < 0.001 vs LPS group; ^$$$^*P* < 0.001 vs LPS + ADSC group. **B** Autophagy in cocultured ADSCs was detected in accordance with the mRFP-GFP-LC3 dual-fluorescence autophagy indicator system under a confocal microscope. ***P < 0.001; ^###^*P* < 0.001. **C** The levels of autophagy-related protein indicators in cocultured ADSCs were detected by WB. ***P < 0.001. **D** The level of autophagosomes in cocultured ADSCs was detected by electron microscopy. Red arrows indicate the autophagosomes. The experiment was repeated triple. Data are shown as means ± SD, **P < 0.01 (determined by unpaired *t* test or one-way ANOVA with Tukey comparisons).

Previous results suggest that ADSCs have an anti-inflammatory function in mouse lungs with ALI and autophagy plays a key role in these processes. To confirm that the anti-inflammatory role of ADSCs on alveolar epithelial cells is direct and autophagy-dependent, mouse alveolar epithelial cells were used (MLE-12). A commonly used murine lung cell line that expresses features of normal type II alveolar epithelial cells [25] was used to construct an in vitro assay. We examined the relative level of cytokine expression in ADSCs and MLE-12 in the in vitro Transwell coculture system. MLE-12 cells were cocultured with ADSCs or Becn-1 knockdown ADSCs and subsequently stimulated with LPS. The concentrations of inflammatory cytokines IL-2, TNF-α, and IFN-γ in the supernatants were measured with an ELISA. The results showed that LPS stimulation induced the secretion of all three inflammatory cytokines. Although ADSCs could suppress the cytokine concentration, interference with autophagy in the cells suppressed this anti-inflammatory function (Fig. 4A). Autophagic flux can also be traced using an mRFP-GFP-LC3 indicator system [26]. Since mRFP is stable in acidic conditions whereas GFP is not, autophagosomes with a neutral pH will be marked in yellow, autolysosomes with the acidic pH will be indicated in red under a fluorescent microscope. The results confirmed that autophagy activity (free yellow spots in cytosol) was high in ADSCs (the blue structure was nuclear stained with DAPI). Furthermore, the number of autophagosomes and autolysosomes (free red spots) was significantly reduced in ADSCs with Becn-1 shRNA (Fig. 4B). The WB results also showed that the level of autophagy protein markers, Atg3 and LC3-II, were decreased following the knockdown of Becn-1. In addition, the level of autophagic cargo adapter p62 was increased (Fig. 4C). The observations of autophagy (indicted by red arrows) in ADSCs or Becn-1 knockdown ADSCs were consistent under an electron microscope (Fig. 4D). These data suggest that ADSCs have a direct anti-inflammatory effect on alveolar epithelial cells. ADSC autophagy is required for this anti-inflammatory function.

### mTOR signaling maintained autophagy in ADSCs

**Fig. 5:**
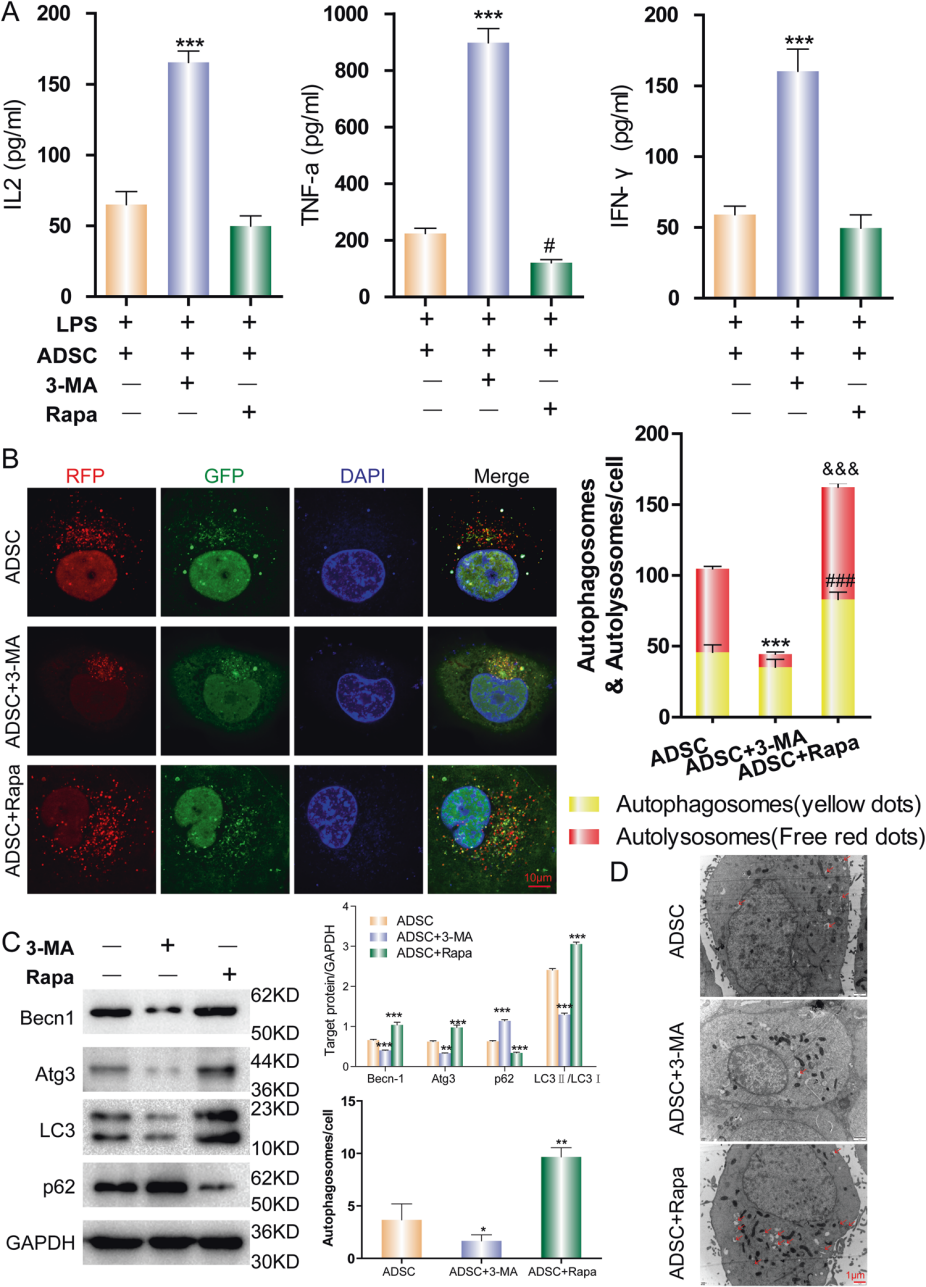
The underlying mechanism of autophagy regulation in ADSC. ADSCs were cultured in vitro and treated with a type III PI3K inhibitor 3-mA or mTOR inhibitor (rapamycin). MLE-12 cells were cocultured with treated ADSCs via a Transwell system and stimulated with LPS. **A** The level of inflammatory cytokines IL-2, TNF-α, and IFN-γ in the supernatants of MLE-12 cells cultured with different groups of ADSCs were detected by ELISA. ***P < 0.001. **B** Autophagy in the different groups of ADSCs was detected using an mRFP-GFP-LC3 dual-fluorescence autophagy indicator system under a confocal microscope. ***P < 0.001; ^###^*P* < 0.001; ^$$$^*P* < 0.001. **C** The levels of autophagy-related protein indicators were detected by WB. ***P < 0.001. **D** Autophagosomes were detected by electron microscopy. Red arrows indicate the autophagosomes. The experiment was repeated triple. Data are shown as means ± SD, *P < 0.05, **P < 0.01. (determined by unpaired *t* test or one-way ANOVA with Tukey comparisons.).

Discoveries over the last decade have demonstrated that the mammalian target of the rapamycin (mTOR) signaling pathway serves as a central regulator of cell metabolism, growth, proliferation, and survival [27]. The mTOR pathway is activated during various cellular processes and its function has been implicated in multiple diseases, including tumor formation, angiogenesis, insulin resistance, adipogenesis, and is deregulated in pathological conditions (e.g., cancer and type 2 diabetes). Since the central inhibitor of autophagy is mTOR, we hypothesized that mTOR regulates ADSC autophagy and the relative anti-inflammatory activity. To suppress or enhance mTOR activity, ADSCs were cultured in vitro and treated with type III phosphatidylinositol 3-kinases (PI3K) inhibitor 3-Methyladenine (3-MA) or the mTOR inhibitor, rapamycin. MLE-12 cells stimulated with LPS and cocultured with 3-MA-treated ADSCs failed to control the level of inflammatory cytokine expression (Fig. 5A). Since the autophagy activity of ADSCs is already at a high level, the addition of rapamycin to ADSCs slightly increased its anti-inflammatory efficacy (Fig. 5A). The consistent inhibitory effect of 3-MA or the promotive effect of rapamycin to the cocultured ADSCs were confirmed with an mRFP-GFP-LC3 dual-fluorescence autophagy indicator system, WB, and electron microscopy (Fig. 5B–D). These results indicate that autophagy in ADSCs have an anti-inflammatory effect on alveolar epithelial cells. Autophagy of ADSCs is required for this anti-inflammatory function.

## Discussion

For decades, extensive pathophysiology research has not helped decrease the mobility and mortality of ALI patients. Thus, a method of deciphering a critical therapeutic strategy to summon variously related resources to achieve their effects is both scientifically and clinically important. Pioneering research into cellular therapy for tissue damage have found that bone marrow stromal cells displayed a natural wound-healing response, and even played a key role in tissue repair [28]. MSC transplantation has been widely tested for safety and efficacy in the clinical treatment of various diseases, spurring an active evaluation of their potential for ALI treatment [29, 30]. Our study has evolved around the effect-mechanism concern as a universal suspect versus the issue of local context challenge, which helps us to line out our limitations and required future efforts.

Adipose-derived MSCs have emerged as a novel source of adult stem cells for the treatment of diseases related to tissue damage due to its easy accessibility and expandability. Related studies have implied that these therapeutic properties of ADSCs are carried out by the cell’s capacity to differentiate into functional target cells to protect against tissue injury, as well as secrete a series of factors that promote tissue repair [31]. Based on our results, our study further revealed the role of ADSCs in the protection of tissue injury and the modulation of inflammation. Our results further suggest that the anti-inflammatory role of ADSCs is maintained by autophagy. However, we also recognize the necessity for future efforts to focus on the underlying mechanism, assuming that there is a possible association between anti-inflammation and autophagy. Our study provides further speculation that there might be an effect of the secretion of active substances (e.g., paracrine cytokines and exosomes) [32] on the maintenance we have attributed to autophagy.

By co-culturing mouse MLE-12 alveolar epithelial cells in vitro, we verified that ADSCs have an independent anti-inflammatory function without the help of other cells that might otherwise be assumed to participate in the process. We also showed that the anti-inflammatory role of ADSCs is dependent on mTOR signaling maintained by autophagy. Considering that ALI pathogenesis is primarily caused by injury to both the vascular endothelium and alveolar epithelium [1], our study yields important evidential support to the development of ADSC transplantation for the treatment of human ALI; however, our evidence does not extend beyond the effect-mechanism association involving autophagy. In particular, when considering that pathological changes in human ALI (e.g., neutrophilic alveolitis, injury to the alveolar epithelium and endothelium, hyaline membrane formation, and microvascular thrombi), potential minor influences of other cells in the lung tissue should be considered before the clinical therapeutic efficacy can truly be justified.

It is important to note that this is the relevance of our animal model to human cases. Previously established major causes of human ALI include lung infection, aspiration, sepsis, multiple trauma, and shock [1]. Mouse models of experimental lung injury based on different types of risk factors have been used to investigate the underlying mechanisms [33]. LPS is recognized as the most important mediator of gram-negative bacteria-induced sepsis, and the systemic administration of LPS in mice is considered to be the classic approach for modeling the consequences of bacterial sepsis. This is precisely why an LPS-induced ALI model was selected for the study of the therapeutic effect of ADSCs. However, for otherwise induced ALI, our animal model may not be as appropriate in terms of pathogenicity. In an LPS-induced ALI model, the capillary endothelium represents the initial site of tissue injury following intravenous LPS administration. Moreover, LPS-induced tissue injury appeared to be related to apoptosis [34]. Cell death and tissue inflammation always form a positive feedback cycle during tissue injury [2]. Considering such a positive feedback cycle, the protective effect and anti-inflammatory role of ADSCs on ALI might be further related to the suppression of epithelial cell death.

In addition, our survey of the literature has also shown that the use of cell-free therapies based on ADSCs, including cell-derived conditioned medium and cell-released extracellular vesicles, provide alternate therapeutic strategies for ALI that are described as ideal in terms of the risks for iatrogenic tumors [35, 36]. Thus, we also recommend further studies to compare cell transplantation and cell-free therapies as alternative approaches to ALI.

## Conclusions

Our findings demonstrate the effect of ADSCs on ALI, especially in alveolar epithelial cells, is dependent on autophagy maintained by mTOR, which provides novel targets and an experimental basis for ALI treatment.

## Materials and methods

### Animals

Mice were purchased from Hangzhou Zishi Laboratory Animal Science and Technology Co., and maintained under pathogen-free conditions. All procedures were approved and conducted under the Guideline for the Institutional Animal Care and Use Committee of Zhejiang University.

### Chemicals and reagents

LPS (L2630) and 3-Methyladenine (5142-23-4) were obtained from Sigma-Aldrich (St. Louis, MO, USA). Rapamycin was purchased from Selleck (s1039, Shanghai, China). Antibodies used for WB were all obtained from Cell Signaling Technology (Danvers, MA, USA). Flow cytometry antibodies were all purchased from Thermo Fisher Scientific (Cambridge, MA, USA). ELISA kits for cytokine detection were acquired from Abcam (Cambridge, MA, USA).

### ADSC isolation and culture

ADSCs were obtained from the inguinal adipose tissue of male C57BL/6 mice digested with 0.075% collagenase I in a 37 °C incubator for 60 min with shaking every 5–10 min. Collagenase I digestion was terminated after 60 min in high glucose Dulbecco’s Eagle medium (DMEM) containing 10% FBS. Following centrifugation (1700 rpm/min, 8 min), the supernatant and undigested fat were removed. The cell pellets were resuspended with DMEM containing 10% FBS and antibiotics, centrifuged (1600 rpm/min for 7 min), and the supernatant and undigested fat were removed. Cell pellets were resuspended in DMEM containing 10% FBS and antibiotics, centrifuged (1600 rpm/min, 5 min), supernatants were removed along with the undigested fat, and the cell pellets were resuspended in DMEM with 10% FBS and antibiotics. The cells were cultured in suspension in a 25-cm^2^ flask at 37 °C in a 5% CO_2_ incubator. The cultured cells were observed under an inverted phase contrast microscope.

### ADSC characterization

The ADSC profile was confirmed through membrane receptor phenotyping and cell differentiation assays. Stem cell positive markers (CD29 and CD44 and negative markers (I-A/I-E and CD31) were determined by flow cytometry. The pluripotency of the ADSCs was determined by osteogenesis and adipogenesis. Osteogenesis was induced in low glucose DMEM containing 10% FBS, 0.1 μM dexamethasone, 0.2 mM ascorbic acid, and 10 mM beta glycerol phosphate for 24 days. Osteocyte calcium deposits were stained with Alizarin Red. Cells were fixed with 95% ethanol for 10 min, incubated with 0.1% Alizarin Red-Tris-HCl (PH 8.3) 37 °C for 30 min, and washed until clear. Adipogenesis was induced in high glucose DMEM medium supplemented with 10% FBS, 1 μM dexamethasone, 0.5 mM isobutyl methylxanthine, 10 μg/mL insulin, and 100 μM indomethacin for 18 days. The adipocyte lipid was stained with Oil Red. Saturated oil red O stock solution was diluted at 3:2 (Oil Red O: distilled water). Samples were washed with 60% propanol, incubated with Oil Red O (Solarbio Science & Technology Co., Ltd, Beijing, China) for 10 min, and counterstained with Mayer hematoxylin before being washed.

### ALI model

An LPS-induced ALI model in mice was established by an airway administration of LPS (500 µg/50 µL per mouse) in mice, and 2 h later ADSCs or PBS (5 × 10^5^ cells/40 µL) were injected to mice with ALI via the tail vein. At 24 h after modeling, lung microvascular permeability was detected according to the Evans blue method (*n* = 10 per group). The lung dry-to-wet weight ratio was detected by weighing the lungs (*n* = 10 in each group). The extent of lung injury and inflammatory cell infiltration were determined by hematoxylin and eosin (H&E) staining (*n* = 10 per group). The number and classification of nucleated cells were determined from specimens retained from the bronchoalveolar lavage fluid (BALF) (*n* = 10 per group). The total protein content in the BALF was determined using a BCA assay (*n* = 10 per group). The level of inflammatory cytokines in the BALF was measured by an enzyme-linked immunosorbent assay (ELISA) (*n* = 10 per group). The survival of mice in each group was calculated by observation (*n* = 20 in each group, weight loss >20%).

### Evans blue

Evans blue (EB) (30 mg/kg) was injected into the external jugular vein 2 h before the end of the experiment. After thoracotomy, the lung was perfused with phosphate-buffered saline containing 5 mm ethylenediamine tetraacetic acid, and the whole lung was excised, sucked dry, weighed, and quickly frozen in liquid nitrogen. Take the right lung homogenate and put it into phosphate buffer (1 ml/100 μg tissue), incubated with two volumes of formamide for 18 h at 60 °C. The optical density of the supernatant was determined by spectrophotometry (620 nm). The EB concentration exuded from lung homogenate was calculated according to the standard curve and expressed in micrograms of Evans blue dye per gram of lung tissue.

### Measurement of wet/dry ratio

The left lung was harvested, weighed, and dried in an oven at 60 °C for 24 h. After dehydration, the lung tissue was harvested and weighed again. The wet/dry ratio was then calculated to assess the severity of the lung injury.

### BALF analysis

BALF was collected by injecting 3 × 1 ml PBS into the lungs. Total protein in BALF was performed using BCA kit (Beyotime, China) and the levels of IFN-γ, IL-2, and TNF-α in BALF were determined using ELISA kit according to the manufacturer’s instructions.

### H&E staining

Lung tissues were fixed in 10% formalin for 24 h. The fixed tissue was rinsed, dehydrated (different concentrations of ethanol), and finally embedded in paraffin. After that, the tissues were sectioned (5 μm) and stained with hematoxylin and eosin.

### Flow cytometry

Surface markers of ADSCs were identified by using flow cytometry. ADSCs were stained with antibodies (CD31, CD29, CD44) at 4 °C for 30 min in the dark. Stained cells were analyzed by a FACSCalibur flow cytometer (BD Biosciences) (San Jose, CA, USA). Data were analyzed using FlowJo version 5 (Ashland, OR, USA).

### Transfection of target cell lines

Lentiviral shRNA virus was bought from Hanheng Company (Hanheng Biotechnology Co. Ltd. Shanghai, China). ADSCs were passaged to 40% confluence and cultured for 24 h. Virus-containing medium was added to the cells. Twenty-four hours later, the viral particle-containing medium was removed and replaced with fresh medium. From days 4 to 10, the medium with puromycin was replaced, and the cells were evaluated for cytotoxicity under a microscope. Finally, the cells were collected for further experiments.

### ADSC MLE-12 transwell coculture

Mouse-derived ADSCs were isolated and cultured in vitro. Transwell chambers were used to coculture ADSCs and MLE-12. ADSC plus medium (high glucose DMEM containing 5% FBS) was placed in the lower chamber, and MLE-12 was placed on the upper chamber. Either 3-MA (5 mM) or Rapamycin (5 μM) was added to the ADSCs in a transwell was 2 h before MLE-12 stimulation. For MLE-12 stimulation, 10 μg/mL LPS was added to the cells for 24 h. The cocultured supernatant was collected from the upper chamber, and inflammatory cytokines were measured by an ELISA. ADSCs were harvested, and the ADSC ultrastructure and number of autophagosomes were detected by electron microscopy. The expression and distribution of autophagy-related proteins in ADSCs were determined by immunofluorescence. ADSC proteins were collected, and a western blot was used to detect changes in mTOR signaling molecules and autophagy-related proteins.

### Western blotting analysis

Western blotting was performed to measure the protein levels of autophagy-associated genes in mouse epithelial cells. Total proteins were acquired by lysing cells with RIPA Lysis Buffer. Protein concentrations were determined using a BCA protein assay kit. Protein (40 μg) was separated by a 10% SDS-PAGE gel and transferred to a polyvinylidene fluoride membrane. After blocking with 5% skimmed milk for 2 h, the membrane was incubated with primary antibodies overnight at 4 °C. The primary antibodies included anti-LC3 (1:1000), anti-P62 (1:1000), anti-Beclin-1 (1:1000), anti-ATG3 (1:1000), anti-GAPDH (1:1000). The membrane was then washed three times with TBST and incubated with horseradish peroxidase-conjugated anti-rabbit or anti-mouse antibodies (1:2000) at room temperature for 2 h. Then the expression of the protein was detected by chemiluminescence reagent.

### Detection of autophagy via mRFP-GFP-LC3 dual fluorescence

To express the dual fluorescence of mRFP-GFP-LC3 in ADSC cells, a mRFP-GFP-LC3 lentivirus was added to the cells in each group for 48 h. Laser confocal microscopy was used to monitor the changes in red and green LC3 expression in the cells. A total of 300 cells were randomly selected to be photographed. The experiments were repeated three times.

### Detection of autophagy via transmission electron microscopy

ADSC cells were fixed in 2.5% glutaraldehyde. The samples were prepared following the standard methods using a TECNA1 10 transmission electron microscope (FEI, Hillsboro, OR, USA) for the image analysis.

### Statistical analysis

Data were obtained from at least three separate experiments performed in triplicate. All results were expressed as the mean and standard deviation (mean ± SD). Comparisons between the two groups were performed with an unpaired *t* test. Differences between multiple groups were analyzed by a one-way ANOVA with Tukey comparisons. A *P* value < 0.05 was considered to be statistically significant. To analyze the survival rate, a log-rank (Mantel–Cox) Test was performed. Data were considered to be statistically significant if *P* < 0.05. Statistical analysis was performed using the Prism software program (GraphPad Software, San Diego, CA, USA).

## Supplementary information

certificate

Original Data File

## Data Availability

All data generated or analyzed during this study are included in this published article.
