# Supplementary material for: Protection of adipose-derived mesenchymal stromal cells during acute lung injury requires autophagy maintained by mTOR
Source: Cell Death Discov. 2022 Dec 5;8:481. doi: 10.1038/s41420-022-01267-z (PMC9722689; doi:10.1038/s41420-022-01267-z)
Supplement: Supplementary file 1 — certificate [file 41420_2022_1267_MOESM1_ESM.pdf]

www.elixigen.com

08/18/2022

Editorial Certification

This document certifies that the manuscript titled "Protection of adipose-derived mesenchymal stromal cells during acute lung injury requires autophagy maintained by mTOR" was edited for proper English language, grammar, punctuation, spelling, and overall style by one or more of the highly qualified native English speaking editors at ELIXIGEN.

Neither the research content nor the authors' intentions were altered in any way during the editing process. Documents receiving this certification should be English-ready for publication - however, the author has the ability to accept or reject our suggestions and changes. To verify the final ELIXIGEN edited version, please contact ELIXIGEN at support@elixigen.com.

*We are NOT responsible for any errors in the added content to our revised version after this date.

ELIXIGEN USA
EXCELLENCE BEGINS WITH ELIXIGEN
