# Supplementary figures and images for: Protection of adipose-derived mesenchymal stromal cells during acute lung injury requires autophagy maintained by mTOR
Source: Cell Death Discov. 2022 Dec 5;8:481. doi: 10.1038/s41420-022-01267-z (PMC9722689; doi:10.1038/s41420-022-01267-z)

Figure 4


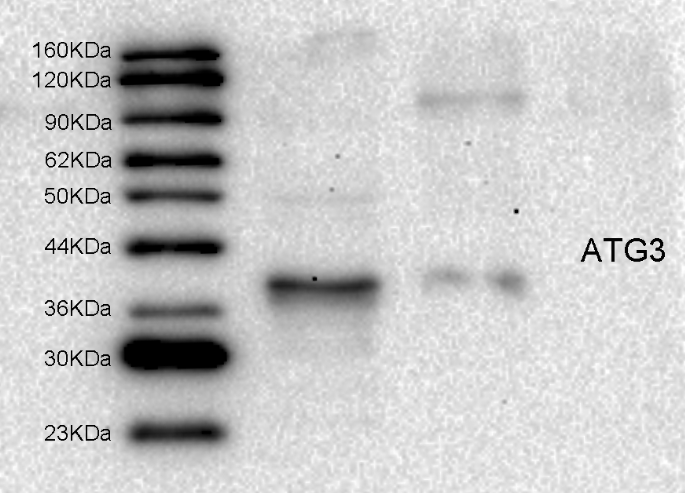


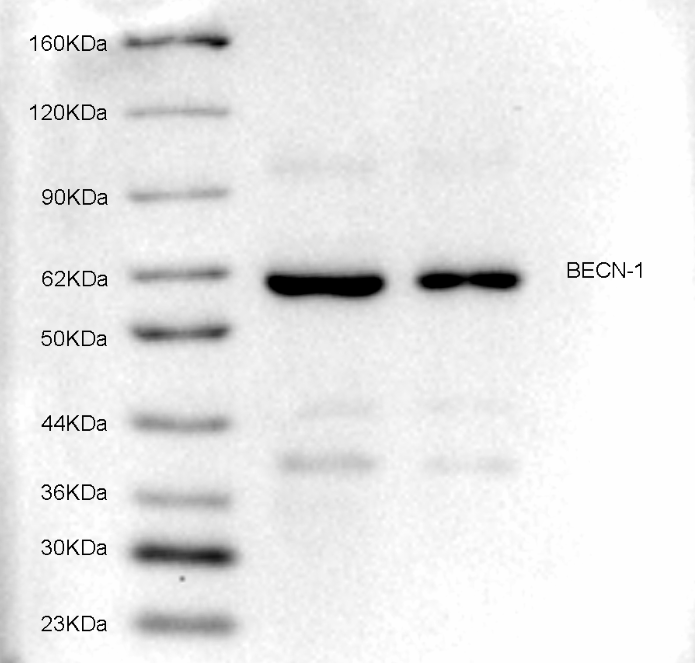

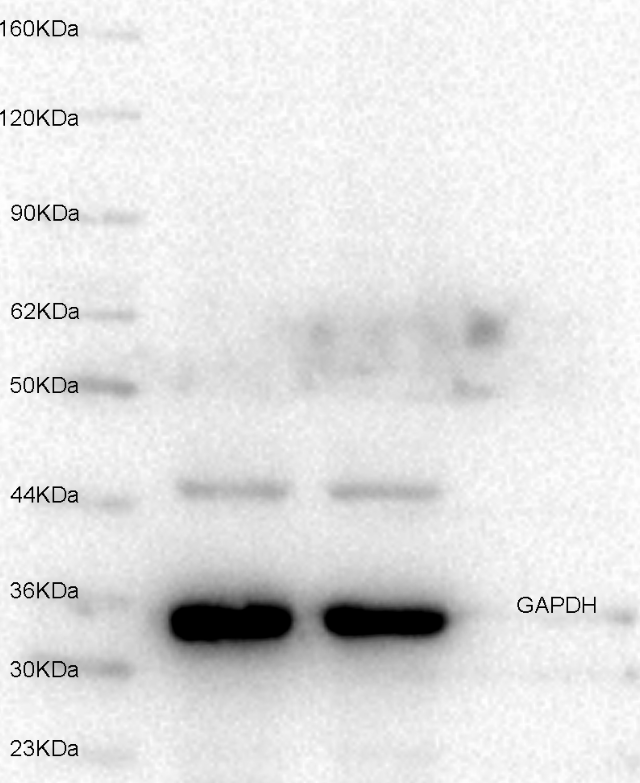


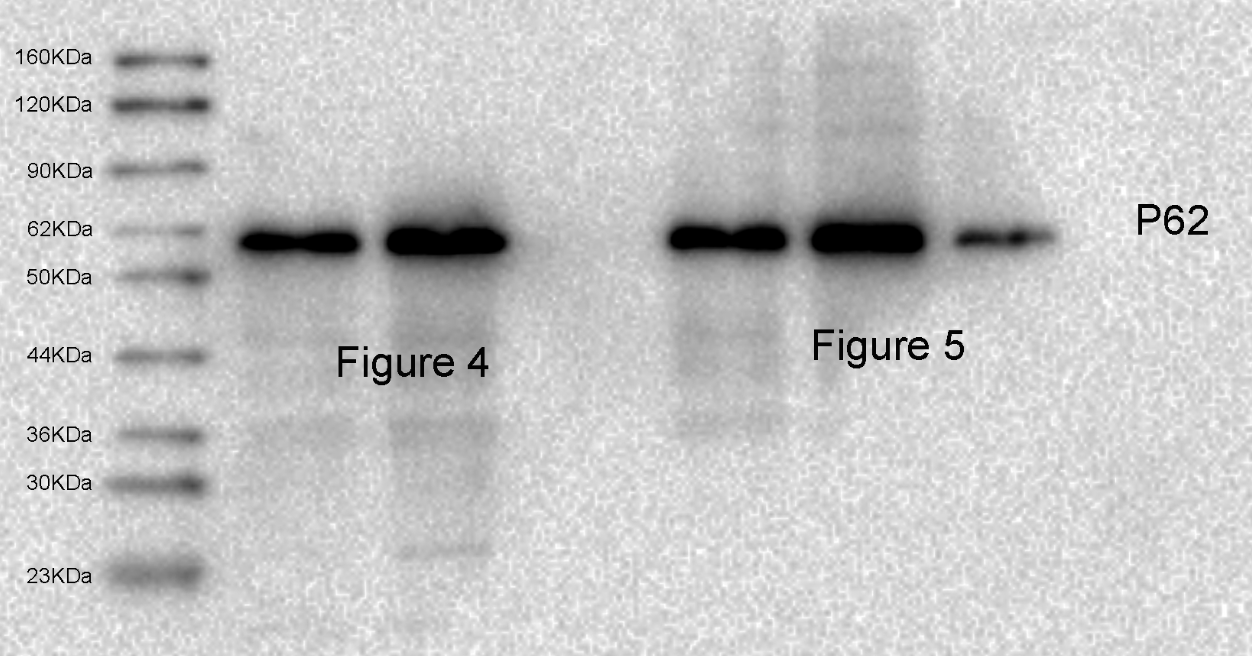


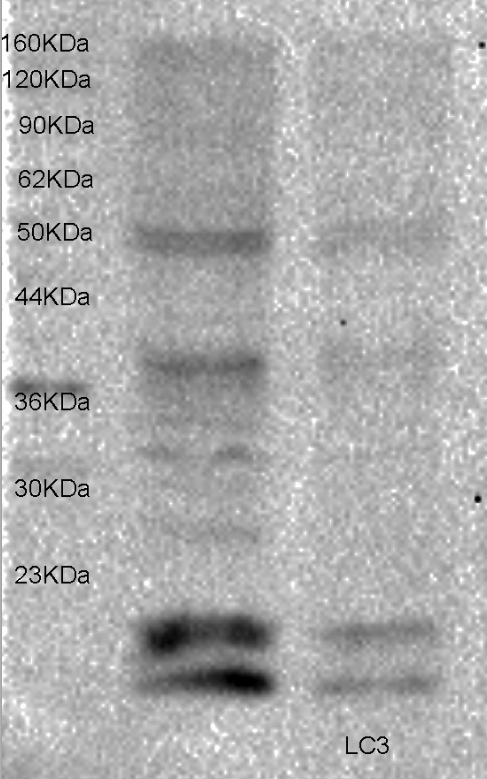


Figure 5


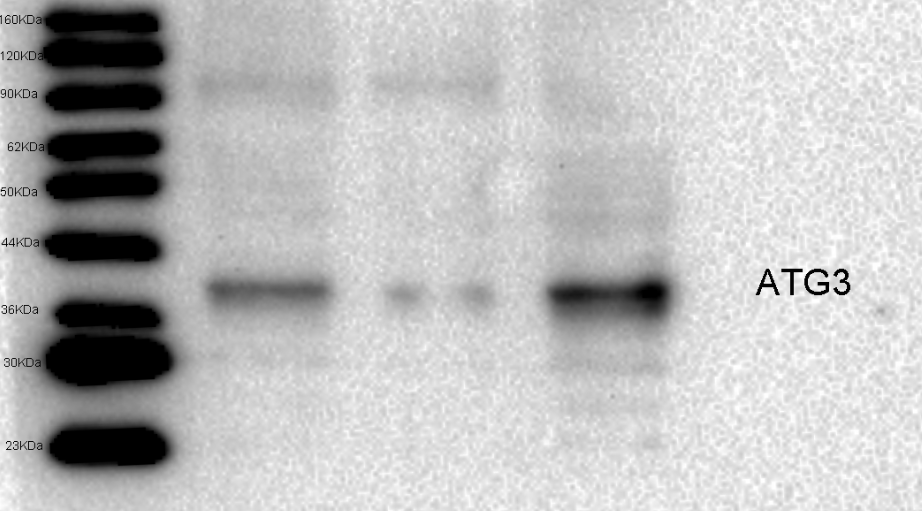


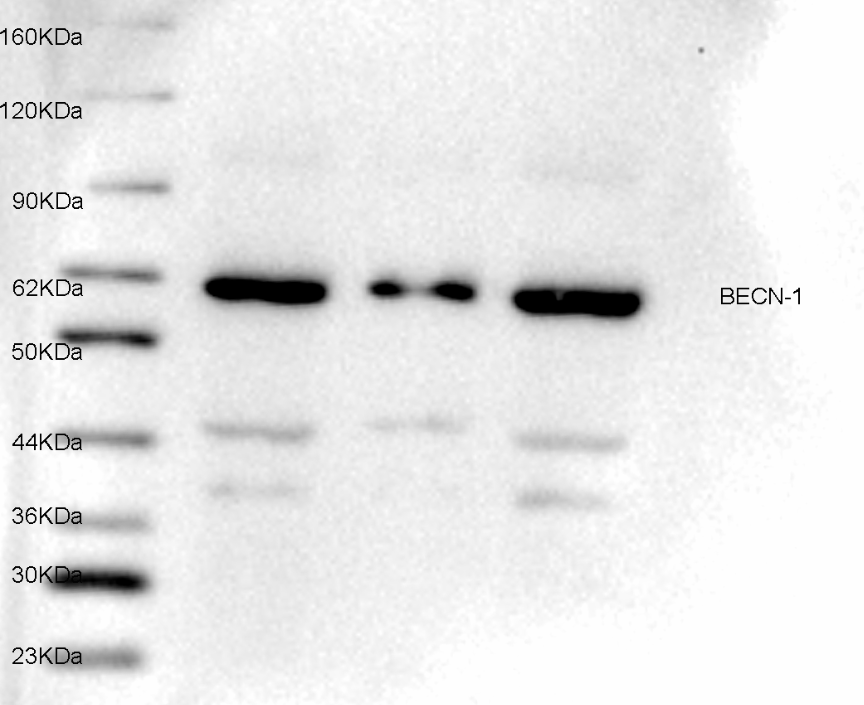


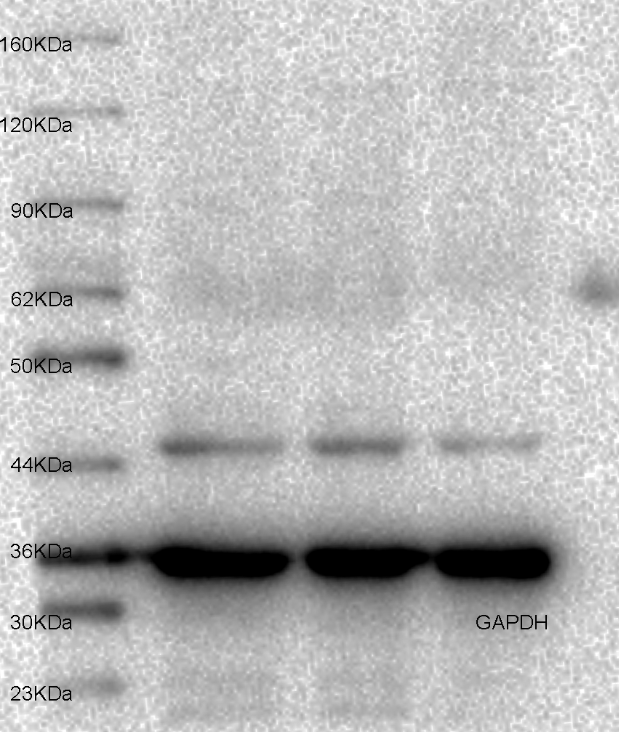

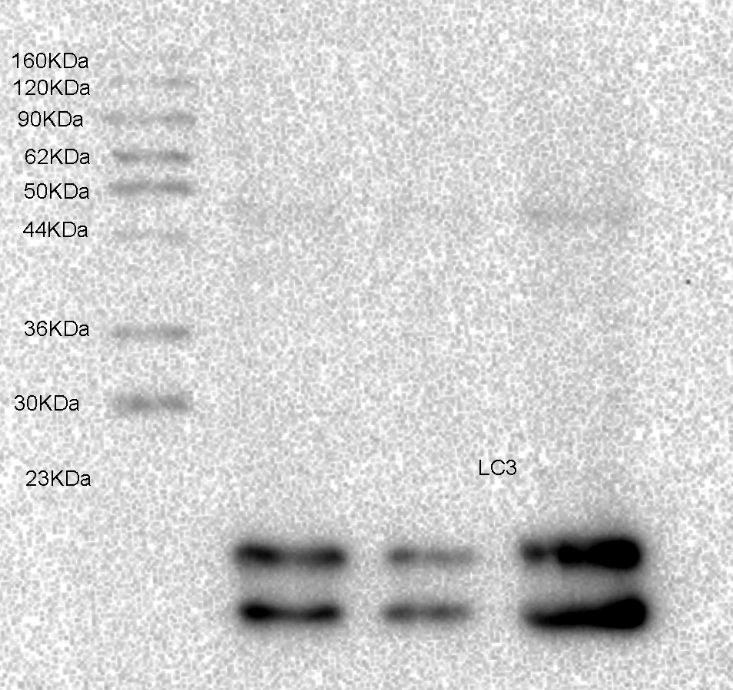


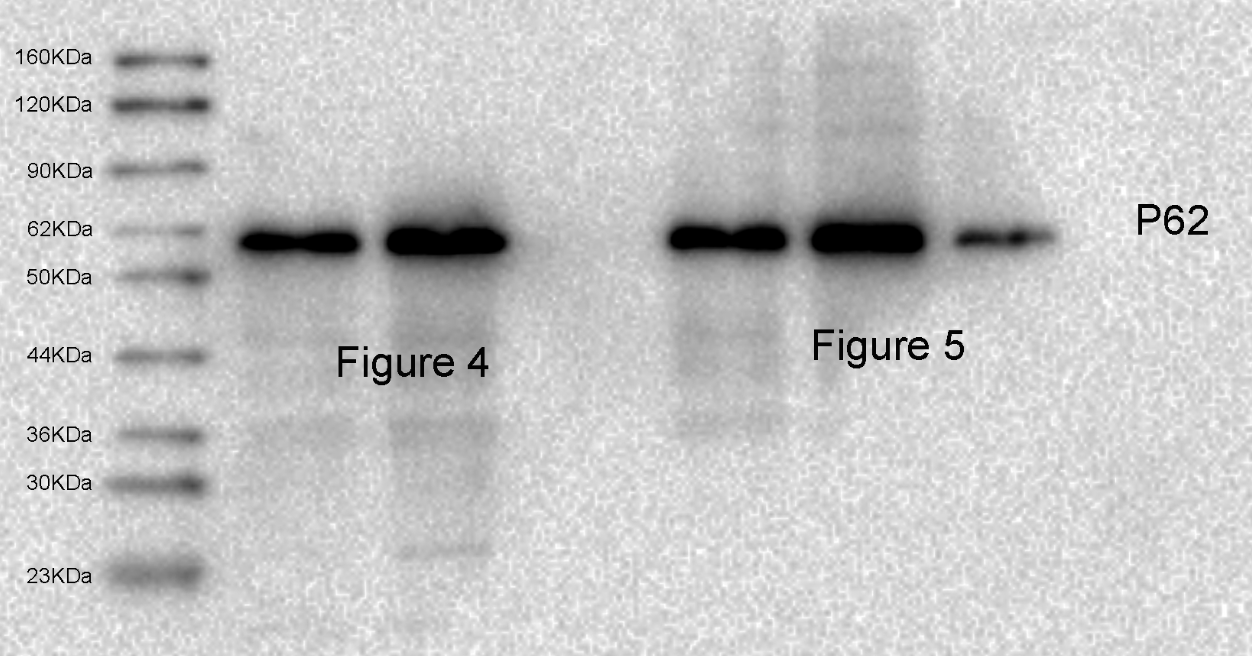

Supplement: Supplementary file 2 — Original Data File [file 41420_2022_1267_MOESM2_ESM.doc]
